# Content Analysis and Qualitative Study of Hemodialysis Patients, Family Experience and Perceived Social Support

**DOI:** 10.5812/ircmj.13748

**Published:** 2014-03-05

**Authors:** Nader Aghakhani, Farkhondeh Sharif, Zahra Molazem, Hosein Habibzadeh

**Affiliations:** 1 Department of Nursing, Faculty of Nursing and Midwifery, Shiraz University of Medical Sciences, Shiraz, IR Iran; 2 Department of Mental Health Nursing, Community Based Psychiatric Care Research Center, Faculty of Nursing and Midwifery, Shiraz University of Medical Sciences, Shiraz, IR Iran; 3 Department of Nursing, Faculty of Nursing and Midwifery, Urmia University of Medical Sciences, Urmia, IR Iran

**Keywords:** *Qualitative Research*, *Social Support*, *Content Analysis*

## Abstract

**Background:**

Various treatments such as hemodialysis prolong the life of chronic renal failure disease patients who must tolerate many physical, emotional, social and economic difficulties. Therefore, social support is considered as a vital area of investigation for such patients.

**Objectives:**

In this qualitative research, a grounded theory approach was used and written as a content analysis form to study hemodialysis patients and family experience of perceived social support.

**Patients and Methods:**

Three nurses, 4 general practitioners, a specialist and two family members who participated were interviewed from April to September 2012 in Urmia, Iran. Interviews were guided to divulge the perception of changes in their lives, needs for social support for disease complications, and the type of treatment process. Purposive sampling continued up to data saturation. Data analysis was performed based on Strauss and Corbin Method. Constant comparison analysis was performed until data saturation.

**Results:**

The research results are shown in 3 steps. In the first step, 113 categories and four main themes from 993 first codes were explored. Social support was explored based on the implications of five general themes including “Perceived Threats Caused by Disease Complications”, “Searching for Social Support”, “Accessible Social Support”, “Beliefs and Values”, and “Perceived Social Support”.

**Conclusions:**

The core variable of our research is acceptance of the reality of the conditions caused by the disease. The research finalized our knowledge about patient problems regarding social support and revealed many problems of supporting patients by Health Team Members, family members and organizations. The findings suggest that individual aspects of patient experiences must be considered if social support is to be given and Healthcare Providers have to facilitate positive health services.

## 1. Background

Chronic Renal Failure is a disease where the resultant kidney failure requires artificial means of excreting waste materials such as peritoneal dialysis or hemodialysis (1). Limitations caused by the disease significantly impact the patients’ beliefs about the illness and sense of control, leading to lack of coping and adjustment (2). Patients face many challenges due to their condition which may leave them feeling fatigued and depressed in hemodialysis (3). It may affect survival of patients and lead to changes in the their family roles and ability to work, including feelings of loss of control and fear of death (4). Patients receiving hemodialysis develop a new identity. This new emotional/psychological state indicates patient dependence on machinery, medication, and Healthcare Providers (5). Dialysis patients suffer from problems, however, they need stable social circumstances including job, income, health insurance and social welfare services (6). Access to social support amongst patients undergoing hemodialysis from the spouse, family members, friends, colleagues or the community is important for better health outcomes and survival (7). It may be helpful if the Health Care Team satisfies these needs in the patient. Social support may have important clinical benefits for the patient population in the promotion or improvement of support networks (8).

Many studies have been carried out on psychosocial problems among patients undergoing hemodialysis. This qualitative study was conducted to identify the main concerns and needs of patients through the analysis of their experiences during hemodialysis to bridge the gap in this aspect of nursing care. No qualitative studies exist on the social support of patients in Iran.

## 2. Objectives

The aim of this study was comprehension of the participants’ perceptions in order to provide Healthcare Professionals ways of planning helpful strategies for their patients.

## 3. Patients and Methods

In this study, a grounded theory approach was used to describe hemodialysis patients' experiences of their perceived social support that results in the development of explanatory theories for human behavior in a social context and areas in which little understanding is available (9). Content Analysis Methodology was used for studying the subject matter of the research. Content Analysis Methodology was developed as a technique to refute existing hypotheses. It was used for formulating “Grounded Theory”, when referred to as “The Constant Comparative Method of Qualitative Analysis” by Glaser (10). The method of content analysis enables the researcher to include large amounts of textual information and systematically identify its properties, such as the frequencies of most used keywords by locating the more important structures of its communication content. The mentioned quantities of textual information must be categorized to provide a meaningful reading of the content under scrutiny. This pilot study was conducted before starting actual data collection. Key informants were 22 patients who were treated by hemodialysis in hospitals of Urmia University of Medical Sciences, 3 nurses, 4 general practitioners, a specialist and two family members who participated in the study from April to September 2012 in Urmia, Iran. The pilot study was carried out to test the acceptability of the questions and responses of the participants. Adjustments were made to the questions. The inclusion criteria were selected individuals with specific knowledge or expertise of the issue being investigated and the exclusion criteria were individuals who did not want to participate in the study. Snowball sampling was used as purposive sampling regarding objectives of the study and thereafter theoretical sampling to emerge data. The researcher started by identifying individuals (at least two) who were relevant to the study. First, some questions were prepared based on the goals of the study (e.g. explain your experiences about you /your patient’s problems). Other phrases such as “What do you mean?” “How?” were also used. All the interviews were recorded and then transcribed verbatim. Data collection and analysis were performed simultaneously. Each interview began with a preamble setting the parameters of the interview (length and audio taping). The research environments were hemodialysis wards to which the patients were referred.

After interviews, each transcript was read line by line and then coding was carried out in 3 stages as recommended by Strauss and Corbin (9). After each interview and throughout the data analysis, the researcher transcribed memos. The basic concepts were extracted in the open coding stage. Similarities and differences were explored by constant comparison, data was clustered, and eventually the categories emerged from well-developed concepts. Four criteria of transferability, dependability, credibility and conformability were used to increase rigor (11). With regard to the fact that entry into their search environment in the present study started from April to September 2012, the researcher believed that he had sufficiently engaged the research environment. Data combination was employed in the present study, and the following results emerged.

### 3.1. Ethical Consideration

All Ethical issues (such as informed consent, conflict of interest, misconduct, co-authorship, double submission, etc.) have been considered carefully. Ethical permission (N0.188) for the study was obtained from the scientific committees of the participating hospitals on August 20, 2012. The ethical principles of autonomy, beneficence, non-maleficence, fidelity and confidentiality have been described relevant to the proposed study. A letter of invite/explanation has been provided for participants along with a consent form. Participants will be advised that they have the right to withdraw from the study at any time.

## 4. Results

### 4.1. Emerging Themes

About 993 initial codes were gathered from all the interviews. Selection criteria included:

1. > 18 years of age
2. Ability to speak Farsi language
3. Diagnosed with ESRD more than 6 months
4. Dialysis treatment at least for a year

The following five themes emerging from the data highlight the factors that impact the study population. The participants’ experience and remarks formed five themes of “Perceived Threats Caused by Disease”, “Seeking Social Support”, “Accessible Resources for Social Support”, “Beliefs and Values" and "Perceived Social Support" plus 18 subcategories. 15 (47%) respondents were female. The participants’ average age was 45.1 years. Because of disease and as complications intensified, the participants perceived that their lives were threatened by hemodialysis. Therefore, they tried to manage themselves. This category, “Perceived Threats Caused by Disease”, is shown by the following aspects:

#### 4.1.1. Perceived Threats Caused by Disease

The individuals’ confusion about the process of perception of apparent threats caused by the disease was clear in the participant narrations. The four subcategories were:

##### 4.1.1.1. Perception of Somatic and Psychological Disturbance of Disease

A participant stated:

“When I referred for a checkup to a clinic, the results horrified me. My God! I would be sick for the rest of my life. I saw my life ending. I felt fear and became depressed”.

##### 4.1.1.2. Fear of Dependency

A participant stated:

“I am disappointed. I had lost my job, my position in my family and health and I had to beg for everything. I had lost my independence. I could not do the things that I did before and I needed somebody to help me”.

##### 4.1.1.3. Threat Caused by Disease

A participant stated:

“Chronic kidney disease has no prognosis. I am afraid of death and disability”.

##### 4.1.1.4. Hemodialysis: An Unpleasant Treatment

A Physician stated:

“There is decrease in blood pressure, cramps occur toward the end of the dialysis procedure, infection, arrhythmia, hemolysis, and hypoxemia are frequent complications of hemodialysis”.

#### 4.1.2. Seeking Social Support Resources

Following the perception of threat and being at risk, patients got anxious and needed physical, psychological, and spiritual support. They decided to appeal to God and ask others for help and support. The 4 subcategories of this concept are as follows:

##### 4.1.2.1. Knowledge of the Present Situation

A participant stated:

“When my disease began, I knew my life was at risk. Nobody was there to give me insight, not even the doctor or nurse who were there to help me”.

##### 4.1.2.2. Gaining Strategies for Social Support

A patient stated: “I lost my health. I suffered a lot. Is there anybody to help me? What can I do? Is there any support for me? Where can I go?”

A participant said:

“Nobody knows about the supportive organizations, I only know about insurance organizations whose social support is insufficient”.

A participant said:

“I am afraid that my condition causes disabilities that prevent support from others. If there is somebody responsible in the ward, we will have fewer difficulties”.

##### 4.1.2.3. Lack of Attention to Social Support

A participant said:

“Nobody sympathizes with our problems here. They work routinely for their salaries, not to solve our problems. You are not important to anybody, unless you are famous or rich or familiar with a doctor here”.

##### 4.1.2.4. Effort for Helping Others

A nurse said:

“Our abilities for helping them are limited. I am very sorry that I cannot do anything for them”.

#### 4.1.3. Accessible Resources for Social Support

##### 4.1.3.1. Family Support

A participant stated:

“I cannot get a job and my father pays my expenses”.

##### 4.1.3.2. Society Support

Some patients had experience of accessible social support and the related difficulties. They reported that the counseling they received from the personnel helped them to cope with and adjust to the reality of the disease, a participant said:

##### 4.1.3.3. Attention to Routine Duties by Health System Personnel

Many participants and their family members believed that the personnel should help beyond their routine duties.

A participant said:

"Last week, I had footache, I told a doctor and nurses, but nobody paid attention”.

##### 4.1.3.4. Communications With Health Personnel

The participants had many concerns about their disease, mainly about its lifetime duration and they believed that they should have communication with personnel for guidelines.

A participant stated his concerns with the following phrase:

“The doctor gave me much medication and the nurses paid me much attention. It would be better, if they gave us guidelines or a booklet, but we got considerable advice from our friends, not the personnel”.

#### 4.1.4. Values and Beliefs

Following the perception of accessible resources for social support, the patients try to accept difficulties based on their values and beliefs.

A participant stated:

“I frequently pray to retain my health for my family’s sake. I accept everything God wills.”

Some had a tendency to admit their disease was a penalty for their sins and prayed to God for forgiveness.

“I trust in God .This condition is intended for me by God”.

#### 4.1.5. Perceived Social Support

Social support is a confirmed multidimensional construct and there is limited understanding of what aspects of social support are important for patients and how much social support may influence their lives after a disease. Individual experiences about perceived social support were apparent in their narrations. The 4 subcategories of this concept are as follows:

##### 4.1.5.1. Familial Consequences

Most of the participants were dependent on their families under the circumstances and were taken to the hospital by them. The majority were alone because their disease was chronic. One of the social problems for the patients is unemployment. Most respondents were faced with being jobless, a source of psychological hinderance, creating anxiety about their future. One family member reported:

“My husband does not have a job and does not have enough money to provide for the family. That’s why there are days we cannot buy his drugs”.

One participant reported that:

“I am obliged to come to the hospital three times a week. How can I work under such conditions? What will happen to me?”

##### 4.1.5.2. Consequences of the Disease

Functional impairment was an important factor in ability to carry on normal activities. The majority of participants indicated that disease contributes negatively to health.

One participant said:

“I feel that I have lost my health after this disease. I cannot do whatever that I used to do in the past. I cannot perform my daily activities”.

##### 4.1.5.3. Hope to Create Positive Changes by Social Support

One participant said:

“If the patient is supported by others, he/she can adapt to the problems”.

## 5. Discussion

This qualitative study created a deeper understanding of interactions and contributions to perceived social support. The characteristics and its treatment methods of End-Stage Renal Disease (ESRD) like hemodialysis are functionally debilitating and can affect the social relationships and activities of daily life (12). The patients undergoing dialysis require different types of social support that affects health through behavioral, physiological and psychological mechanisms depending on the social environment and the severity of the illness (13). Our study of the experiences reported in this study showed that five aspects of perceived social support were mentioned by the participants. Other studies highlight the importance of social support in reducing the incidence of disease that is generally defined in terms of the availability of people who can be relied upon to make a person feel valued (14). Perceived instrumental and expressive types of social support for the patients can cause feelings of worthiness and intimacy within patients (15). Social support provision can be made by emotional means, tangible efforts, information sharing or advice giving by personnel (16). If the feeling of social isolation is not detected, it can cause higher morbidity and mortality (17). In our study, most patients evaluate their social supported status negatively. Another study showed that the patients undertaking hemodialysis are vulnerable and often cannot function fully within society (18). Their ability to perform activities is a strong indicator of survival, a determinant of care giving needs and health care costs, and a factor in medical procedure decision-making (19). Hemodialysis creates considerable stress on patients, changes in family relations and social life and social restrictions or sometimes isolation that must be considered (20). Social support for the patients is a holistic item. However, to provide holistic social support for the patients, other aspects of support such as spiritual, physical, psychological, and social elements of their lives should be considered. Since in the dialysis wards in this research, treatment and care are based on routine work, suitable schedules should be prepared that consider all dimensions of treatment and care in the wards.

Our study showed that living with a chronic illness can greatly compromise the “value systems”. Most of the participants lacked the education and training related to their problems and, therefore, expert staff employment and training courses for patients and their family members can be considered as social support. In this study, the participants mentioned the importance of holistic social support. Also the study reports that family support enhanced the ability of people to keep their positive attitude for the duration of their disease. Therefore, it is necessary to train health professionals to meet the different aspects of need and as the family is one of the most important elements and life is usually dominated by family values (21), the relationship between the members of the family and participants in the care of patients is important in order to modify any feeling of isolation. Also, the results of the study demonstrated the need for training of staff to increase family involvement in care for the patient and to support suitably different kinds of social support (22). Most of the patients wish to be protected by God and seek strength from him. Trust in God has been discussed as the most common coping mechanism when facing stressful situations (23). Because of their problems, the staff should be patient and recognize the patients' physical and cognitive changes which leads to inability in their daily activities (24). The study showed that empathy with patients mostly depended on the characteristics of the health professional (25). Cooperation of the health professionals in supporting patients has a great influence on care. Most of the personnel feel that being with patients sometimes can be the best support for them (26). Therefore, understanding that patients on dialysis require different types of social support has important clinical implications. Health professionals could offer intervention programs to improve social support based on the patient needs, such as recommendations to appropriate programs like self-help groups (27) or educational programs for psychological improvement (28). Unemployment is common in patients; therefore, a job can be a source of social support, and having a job contributes to increased self-esteem (29). Finding a job can help patients. Psychosocial problems can contribute to conflicts between patients and health professionals that could be attributed in part to stressful conditions, including frequent visits and prolonged waiting time in the dialysis unit (30). Health professionals need to assess families to recognize their needs and close partnerships with available social supports and networks to implement family health promotion. They also need to encourage families to use and enhance support networks (31). Public policies, mainly related to the availability of benefits for patients with chronic disease, should be imparted to the patient and his/her family with clarity and in due time. Patients and their families need to feel secure in communication with health professionals. Health professionals should not make precipitated or prejudiced judgments about family members' ability to understand. Available literature confirms conflicts, omissions and misunderstandings between the family and health professionals, similar to data found in this study. Relatives expect health professionals to share information of their patient (32). The core variable of our research is acceptance of the reality of the conditions caused by disease. Our findings show the importance of support for all patients in order to improve their well-being. Support groups should play an important role in planning social support and care which can lead to better health outcomes. An environment must be created to ensure that support groups are better organized to make them more effective in assisting their members. This necessitates the collaboration of governments, non-governmental agencies, organizations, and philanthropic individuals (31). This study can help to promote inter-sectorial support and based on the information in this article, health organization and managers should play a leading role, creating supportive polices. Further research is needed on the subject.
